# Structure prediction in low dimensions: concepts, issues and examples

**DOI:** 10.1098/rsta.2022.0246

**Published:** 2023-07-10

**Authors:** J. Christian Schön

**Affiliations:** Department of Nanoscience, Max-Planck-Institute for Solid State Research, Heisenbergstr. 1, D-70569 Stuttgart, Germany

**Keywords:** structure prediction, low-dimensional systems, energy landscapes, nanotubes, monolayers

## Abstract

Structure prediction of stable and metastable polymorphs of chemical systems in low dimensions has become an important field, since materials that are patterned on the nano-scale are of increasing importance in modern technological applications. While many techniques for the prediction of crystalline structures in three dimensions or of small clusters of atoms have been developed over the past three decades, dealing with low-dimensional systems—ideal one-dimensional and two-dimensional systems, quasi-one-dimensional and quasi-two-dimensional systems, as well as low-dimensional composite systems—poses its own challenges that need to be addressed when developing a systematic methodology for the determination of low-dimensional polymorphs that are suitable for practical applications. Quite generally, the search algorithms that had been developed for three-dimensional systems need to be adjusted when being applied to low-dimensional systems with their own specific constraints; in particular, the embedding of the (quasi-)one-dimensional/two-dimensional system in three dimensions and the influence of stabilizing substrates need to be taken into account, both on a technical and a conceptual level.

This article is part of a discussion meeting issue ‘Supercomputing simulations of advanced materials’.

## 1. Introduction

The development of new devices with technological, medical or biological applications often involves the availability of special materials with extraordinary properties, or at least deep insights into the behaviour of such materials in a variety of environments [1–6]. A traditional road towards achieving these goals follows the experiment: new combinations of educts and controlled variations of synthesis routes together with chemical intuition are employed to generate new compounds in a more or less purposeful way [7]. Once a new material has been synthesized in a reproducible fashion, and its unique characteristic structure has been properly determined, one will determine the physical, chemical and biological properties of the compound. In particular, one will study its stability as a function of the environment ranging from temperature and pressure variations, and the exposure to distinct chemical and biological environments, to the presence of radiation or electromagnetic fields.

This experimental explorative approach will be complemented by the computation of these properties based on first principles or model approaches, with the goal to understand the underlying physical foundation of the material's properties. This allows one to elucidate the connection of the various properties and stabilities with the structure of a material that defines its identity on the most fundamental level, and develop heuristics that can guide the chemical intuition in selecting new promising synthesis targets and synthesis routes; here, modern machine learning using multi-layer (deep) neural networks can also prove to be useful as a black-box-type realization of such a heuristics [8–10].

The starting point for all such computations is the structure of the synthesized material, if known to a sufficiently high degree. But if one wants to establish the long-term stability of the compound, then just performing, e.g. long molecular dynamics or Monte Carlo simulations starting from the synthesized modification of the compound, is often not sufficient, because many relevant phase transformations or ageing processes might not be realized on the time scales and system sizes accessible in the simulation. Instead, estimates of the long-term stability of the material require information about all relevant competing modifications and phases possible in the system, such that the transitions among these polymorphs can be computationally investigated in detail.

Finding these modifications corresponds to the so-called structure prediction of the polymorphs that are capable of existence in a chemical system, i.e. the determination of all the locally ergodic regions *R_i_* on the energy landscape of the system on a given observational time scale *t*_obs_ [11,12]. Typically, the energy landscape corresponds to the potential energy as function of all arrangements of the *N* atoms constituting the chemical system—the state space of the system is then usually ***R****^3N^*—where the energy is computed either on the *ab initio* level or using model potentials or in-between methods such as density functional tight binding (DFTB). The relative thermodynamic stability of these regions is given by their local free energies *F*(*R_i_*)* = −k_B_T* ln(*Z*(*R_i_*)), where *Z*(*R_i_*) is the partition function restricted to the region *R_i_*, and their (kinetic) stability against decay is controlled by both energetic and entropic barriers that combine to yield a survival time *τ*_esc_*(R_i_)* for each region *R_i_*, where *t*_obs_* < τ*_esc_(*R_i_*) for the regions *R_i_* of interest. Of course, a synthesized compound should correspond to one of these stable regions in the configuration space of the chemical system. A very popular application outside of materials development has been the prediction of high-pressure phases, where the potential enthalpy landscape for a variety of pressures is investigated [13–16].

Clearly, such predictions are not restricted to finding additional modifications besides the one(s) already known for a chemical system. We can also study a chemical system, where no compound has been synthesized so far, and thus perform an unbiased prediction of the possible polymorphs for this system, possibly followed by a computation of their properties. Here, it is important to note that we are interested not only in the thermodynamically stable phase but also in metastable modifications, which might have just the right properties and sufficiently high stability to be useful in applications—besides the classical example of diamond, we might think of glasses [17–19] or amorphous semiconductors [20,21], or drug molecules that exhibit different effects in different polymorph structures [22–25].

Being able to perform such an unbiased structure prediction serves as the first step towards the theoretical design of new materials [26]. And while both systematically choosing new synthesis targets and constructing possible synthesis routes have been feasible—to a certain degree, at least—in the field of (small) organic molecules using chemical intuition and heuristic rules of reactions [27,28], such predictions for crystalline compounds [26,29,30] or atom clusters [31–33] have only become possible with the availability of fast computers in the past three decades. Even now, using *ab initio* energies to describe the energy landscape even of a quite small (model) system of interest—say a few dozen atoms per variable periodic simulation cell for crystal-forming compounds—makes a full global exploration of such systems very expensive computationally. Clearly, this reduces the reliability of the predictions in systems where no chemical intuition—perhaps implemented in a neural network trained on ‘chemically similar’ systems—can serve in guiding the search and in judging the quality of the structure candidates that are generated. As a consequence, unbiased structure prediction remains a major task for high-end computational efforts in materials science.

Methods for performing such unbiased structure predictions have been developed and applied for 35 years by now, where the focus of the applications has been on atom clusters in vacuum [31–33], single molecules and biomolecules [34–36] and crystalline compounds [12,26,29,34]. Yet in recent years, low-dimensional systems have become of great interest [37]. In this work, we discuss some of the special aspects that distinguish the structure prediction of low-dimensional systems from those of three-dimensional bulk solids, clusters and single molecules, and present a number of prototypical example systems.

## 2. Concepts and methods in structure prediction of low-dimensional systems

Quite generally, the prediction of structure modifications for low-dimensional chemical systems and of those for finite or infinite three-dimensional systems such as clusters and molecules or crystals follows the same general approach [38]. A useful polymorph is characterized by corresponding to a locally ergodic region on the energy landscape of the system, i.e. for observation times of interest, the system can equilibrate within this region of configuration space but will have only a very small likelihood to transform into an alternative modification, for given thermodynamic boundary conditions [11,12]. In many situations of interest, such ergodic sub-regions *R_i_* are centred around one local minimum of the potential energy (typical for crystalline structures or ordered atom clusters) or around a family of local minima, e.g. the many homotops of an alloy cluster or the many configurations in a solid solution that contribute to its configurational entropy. Here, we also note that entropic aspects are not only relevant for the computation of the local free energy but also play an important role as entropic barriers that help stabilize the region complementing the more familiar energetic barriers [38,39]. As a consequence, the global search algorithms also need to incorporate means to overcome not only energetic barriers but also entropic ones, which are not always amenable to, e.g. clever temperature schedules in the search algorithm.

Thus, many approaches to structure prediction start with a global optimization study of the system, identifying as many of the low-energy local minima—including the global minimum, of course—on the energy landscape as one can. If possible, such a global search is performed employing *ab initio* energy functions, but in many cases, one first finds structure candidates using an empirical potential approximation of the energy followed by a subsequent local optimization of the most promising candidates on *ab initio* level.

From a more technical point of view, we remark that there is an important methodological difference between structure prediction of clusters and single molecules on the one hand and (crystalline) bulk systems on the other hand: since a cluster contains a finite number of atoms, the state space of the system we employ in our analysis consists of all feasible atom arrangements of the cluster. By contrast, due to the essentially infinitely large number of atoms in a bulk material, one cannot represent the complete system in the computer, and one is forced to use rather drastic approximations when searching for structure candidates with only finite computational resources. As discussed in [38], the most commonly used approach is to employ a so-called periodic approximant for the bulk system, i.e. we define the model for the system via the variable cell parameters of a periodic unit cell that contains a finite number of formula units of the system. Of course, this means that we ignore surface effects, and we can only find structure candidates for the system, which are crystalline and do not contain more atoms in the periodic unit cell than the ones we have prescribed in our model.

During the global search, all the atom positions inside the cell and, very importantly, all cell parameters, are free to vary. However, since no information is available regarding the ‘correct’ number of atoms in the cell for the candidates corresponding to low-energy minima of the system, these global searches need to be repeated for as many different numbers of formula units as one can afford computationally, in order to obtain a satisfactory set of structure candidates for the (crystalline) bulk system. Here, one needs to keep in mind that, if we employ *n* formula units for the periodic approximant, we can only find periodic structures with *m ≤ n* formula units in a unit cell for those values of *m* that are divisors of *n*; thus, e.g. using a periodic approximant with *n* = 8 excludes all periodic structure candidates with *m = 3, 5, 6* and *7* formula units per unit cell. We note that, in practice, the number of atoms one can employ is usually too small to incorporate large defects like grain boundaries, i.e. we only obtain candidates for a single crystal with at most (periodic) point defects.

This set of structure candidates is often supplemented by structures that are analogues to structures found in chemically related systems that have been directly extracted from a database [40–43] or generated by an analogy-based algorithm such as a neural network. An overview of some of the most popular algorithms can be found in the literature [38,44].

For low-dimensional systems, the same type of exploration and optimization algorithms can be employed, with straightforward adaptations to lower dimensions as far as the energy calculation is concerned. Here, we usually assume that the interaction between the atoms is the regular interaction in three dimensions, i.e. we do not consider the case of two- or one-dimensional ‘atoms’ that are defined as truly two- or one-dimensional entities with interactions in only two or one dimensions (such systems are more appropriate for abstract models in theoretical physics). Once we have identified promising candidates for locally ergodic regions corresponding to crystalline modifications or solid solutions, we can proceed as in three dimensions and evaluate their local free energies and rank the phases as far as their thermodynamical stability is concerned. Here, we should keep in mind that in low dimensions, the transitions between ordered and disordered phases can take place at much lower temperatures than in three dimensions—the classical textbook example is the one-dimensional-Ising ferromagnet, which is unstable for all non-zero temperatures [45]. An analogous instability can easily occur for binary atom chains; quite generally, in one or two dimensions, the entropic contributions to the free energy can be of a different quality and/or magnitude than in three dimensions, even for otherwise comparable types of systems.

However, an important difference to the three-dimensional systems is the stability analysis of the candidates. Even in an ideal one- or two-dimensional system, the atoms are three-dimensional entities, and the chain or plane of atoms exists in three-dimensional space. Thus, the minimum structures must not only be stable with respect to perturbations in one or two dimensions, i.e. within the configuration space for which the low-dimensional system is defined, but we also must ask about the stability of the (in one or two dimensions) optimized structure when we place it into three-dimensional empty space or on top of a substrate. This kind of embedding of the predicted structures is of central importance in the validity of the prediction process.

Closely related is the, at first sight, technical issue of the compatibility of the periodicity of the substrate on which a (macroscopic) monolayer is deposited or generated, with the periodicity of the perfect two-dimensional pure monolayer system—modelled as a two-dimensional periodic approximant of the essentially infinitely large monolayer—that would be obtained as a global or local minimum as long as we only consider the interactions of the atoms or molecules in the monolayer among themselves, i.e. without the presence of the substrate. As long as we can ignore the substrate, we can freely vary the two-dimensional unit cell of the periodic approximant, such that, within the limitations of periodic approximants, we are able to obtain globally optimized structure candidates for the two-dimensional system. Such a global search of an ideal two-dimensional system will often be the first step in the identification of the structure of monolayers on a substrate, in particular if we can assume that the interactions between the deposited atoms and those located in the surface layer of the substrate are either very weak or do not noticeably vary along the surface. In such a case, one can often approximate the effect of the substrate by a constant attractive force on the atoms and molecules of the deposit, whose only effect is to restrict the movement of the atoms or molecules away from the surface. In such a simplified model of the substrate + deposit system, the substrate's actual structure on the atomic level is irrelevant and no constraints (due to the substrate surface) exist that would limit the variation of the two-dimensional unit cell of the monolayer.

In a second step, one would ‘activate’ the surface–deposit interactions and study the distortions of the candidate structure away from the perfect two-dimensional atom arrangement, together with possible accompanying atom displacements in the (top) substrate layers. In principle, there is no problem as long as we can study the combined system without needing periodic boundary conditions, i.e. if we have enough computer resources to model a realistic (meso- or macroscopic) system. In that case, the only complication is that, for every monolayer structure candidate, we have to test, i.e. locally optimize, many relative (in the *xy*-plane) placements of the candidate with respect to the substrate surface, since we do not know beforehand what will be the best fit of the monolayer candidate onto the substrate. In this context, one should mention that the thickness of the slab in the *z*-direction, which approximates the (usually semi-infinite) substrate, is also a possible variable that needs to be studied when modelling the system, including possible technical aspects such as the appearance of macroscopic dipoles in polarizable ionic substrates.

However, if we have to model a monolayer + substrate system via a periodic approximant, we clearly must respect the fixed periodicity of the substrate, even if we did not include the actual interactions between surface atoms and monolayer atoms in the energy calculations at the global search stage. When we place the candidates we have found in the global search on top of the substrate, to compute the effects of the substrate on the monolayer, this would not be possible, if the periodicity of the monolayer was incompatible with the periodicity of the substrate. Of course, if we have already ascertained that the substrate has no structure guiding effect on the atoms or molecules which we deposit on it, then there would be no problem, and we might save ourselves the effort of optimizing the monolayer + substrate system. But usually, this is not known beforehand, and there will always be some effects due to the spatial distribution of various types of surface atoms.

One way to approach this issue is to find rational approximants, i.e. we construct a supercell of the two-dimensional lattice of the monolayer candidate, which closely matches a supercell of the periodic lattice of the surface, and then squeeze or stretch or twist the monolayer supercell, until it agrees with the surface supercell. This is then followed by a local optimization of the monolayer + substrate system for fixed parameters of the supercell. Of course, one would need to test many placements of the monolayer on top of the substrate, to find the best arrangement of this monolayer + substrate combination. We repeat this procedure for many supercells of a given monolayer candidate and then the whole scheme is repeated for other monolayer candidates found during the global search of the pure two-dimensional system.

If this approach does not yield sensible results, an alternative is to perform the global search with a certain number of atoms or molecules on the real substrate with atom level resolution; this is often the most sensible approach, if we expect that the structure of the substrate surface will strongly guide the structure formation of the deposit. However, we can only use periodic cells that match the substrate periodicity, and, thus, we cannot use the usual highly efficient (in three dimensions) exploration moves of slightly modifying the periodic simulation cell, during the search for structure candidates for the two-dimensional monolayer when the substrate is present on the atom level, since these modified cells would violate the periodicity of the substrate. Instead, we can change the periodic cell in discrete units of the cell vectors of the surface atom lattice. Since this exploration move can involve large jumps in the density of the deposit (atoms/unit cell area), we might want to include the option to add or subtract one or a few formula unit(s) of the deposit as part of the cell change move, such that the density of the deposit does not vary too much during the global exploration; however, this may require the introduction of a chemical potential term into the energy function, greatly complicating the exploration (for more details on such systems, see [38]).

To avoid such complications, an alternative is to perform the global exploration for many fixed simulation cells each corresponding to a supercell of the substrate surface atom arrangement, where for each such cell, we repeat the global exploration runs for different fixed numbers of atoms or molecules. However, it is well known that the imposition of a fixed periodic simulation cell or an external confining boundary during a global search for crystalline structure candidates is a strong and, in general, unphysical restriction on the feasible structures and their energetic ranking [46], even if the simulation cell contains many atoms. Thus, we have to be aware that we might not be able to identify the true periodic arrangements of the bulk two-dimensional system in contact with the substrate, unless either the two periodicities are compatible, the substrate–monolayer interactions are strong enough to enforce the substrate's periodicity on the structure of the monolayer, or the interactions are so weak or indiscriminate that the structure of the monolayer is only determined by the intra-monolayer interactions.

Here, we note that the same kind of constraint on the possible structures appears when studying feasible one-dimensional atom chains on a substrate, or the possible atom configurations on an infinite cylinder. Again, the finite number of atoms inside the periodically repeated piece of the cylinder surface along the cylinder axis, i.e. the variable simulation cell, combined with the constraint of a fixed cylinder radius leads to serious limitations on the optimal structures and their ranking by energy. This is the case even if the length of the cell along the cylinder axis is variable, and we allow the number of atoms on the cylinder surface cell to vary. In particular, we not only find different types of global minimum structures due to different energy rankings but also find that even the set of possible types of qualitatively distinct atom arrangements that correspond to local minima can change when switching to a different radius.

In the case of two dimensions, another alternative route to identifying monolayer structures on a substrate would be to perform the search without imposing any periodic boundary conditions, while still trying to stay within a manageable range as far as the number of atoms or molecules in the deposit + substrate system is concerned. We create an atom ‘cluster’ in the form of a finite (in the *xy*-direction) but very large substrate surface slab (of finite thickness), where we do not impose periodic boundary conditions on the substrate, to allow the atoms of the substrate surface to move away from their equilibrium positions if this helps to minimize the total energy of the substrate + deposit system. However, we usually fix the atoms at the outer edge of the substrate cluster and possibly also those at the bottom layers of this cluster, to represent the semi-infinite extent of the substrate and to prevent unphysical bending of the macroscopic substrate. Next, we place many atoms or molecules belonging to the monolayer of interest on this slab, and let them move freely on the surface—as long as they do not get too close to the edge of the substrate cluster—and find their own (periodic) arrangements. The disadvantage is that these arrangements of the deposited atoms will have a ‘surface’, i.e. a borderline, and thus the number of atoms beyond which the ‘periodic’ arrangement becomes preferred energetically compared with a non-periodic one will often be very large; this is similar to the observation that in many cluster systems the cut-outs from the periodic three-dimensional bulk structure only become the energetically preferred cluster structures once the cluster size exceeds hundreds of atoms. As a consequence, the computational effort can become gigantic, especially if the global search is performed on the *ab initio* level, and even if the global search employs empirical potentials, the local *ab initio* optimizations of the candidates found will be very expensive computationally.

Furthermore, regardless of the issues of how to most efficiently search for the global minimum structure for pure two-dimensional systems on a generic unstructured substrate surface or how to align the periodicity of the monolayer with the periodicity of the substrate, other challenges arise in the realization of two-dimensional monolayers on real substrates when the actual interactions between surface atoms and atoms of the deposit are explicitly included in the energy function at one or all stages of the structure prediction. In the simplest case, the only effect of the substrate is to exert a weak but sufficiently large attractive force on these atoms such that they remain within a flat two-dimensional plane, and the deposited monolayer can be treated as a good representation of the pure two-dimensional system. But in reality, even a perfectly smooth crystalline substrate consists of a periodic arrangement of atoms, which interact individually with the atoms of the monolayer. As a consequence, the arrangement of the atoms belonging to the surface of the slab can induce a preference for a particular structure candidate of the two-dimensional system (possibly with some slight distortion), which does not need to correspond to the global minimum of the pure two-dimensional system. Even more dramatically, the slab's surface can induce a structure for the monolayer, which does not even correspond to any local minimum structure of the substrate-free pure two-dimensional monolayer. Such effects are taken advantage of in atom beam epitaxial growth of thin films of specific crystalline structures [47,48], or during heterogeneous nucleation of particular phases [49,50], and analogous phenomena are involved in stabilizing metastable layered compounds [51,52]. Closely related is the fact that the atom level granularity of the substrate surface will move the atoms of the monolayer slightly out of the perfect two-dimensional plane, i.e. we would need to describe the atom positions in this layer via both the *x*- and *y*-coordinates parallel to the substrate surface and the orthogonal *z*-coordinate. Thus, the monolayer deposit is not perfectly two-dimensional, but exhibits a slight (spatially varying) deformation into the third dimension, with non-trivial consequences for the modelling of the system and the implementation of the global search for structure candidates, which can no longer be restricted to purely two-dimensional atom configurations.

A second major difference to the structure prediction in three dimensions concerns the moveclass of the search algorithm. In general, there are moves during our search on the energy landscape, which imitate the movement of atoms in reality or during molecular dynamics simulations; typically, these moves correspond to small shifts of individual atoms or groups of atoms during the random walk or the deterministic trajectory on the energy landscape. These moves are often complemented by various kinds of jump moves, such as exchanges of atoms that are not neighbours, or the massive mixing of structures during genetic algorithm crossover (parent–children) moves. The latter moves usually do not represent any kind of moves that take place during the real time evolution of the system. Instead, they allow us to jump between basins on the energy landscape, resulting in a faster generation of promising structure candidates; we note that such jump moves are often combined with a local deterministic or stochastic minimization run. Such non-physical moves, which can not only ‘tunnel’ through energy barriers but also might avoid entropic barriers on the energy landscape of the system, can be defined in both three-dimensional and low-dimensional systems and influence the efficiency and completeness of the global search algorithm—depending on the implementation, a possible price for a higher efficiency in avoiding traps on the energy landscape can be the inadvertent elimination of physically relevant local minimum structures [38].

However, if one wants to study the actual movement and structure formation of monolayers of atoms or molecules on a realistic surface with atomic resolution, e.g. to gain insights into the kinetics of the growth process of ordered phases, or if the goal is to determine the kinetic and thermodynamic barriers stabilizing a given modification, we face a different challenge. In this case, the random moves cannot be restricted to two dimensions only (e.g. to the *xy*-plane), because the optimal positions of the atoms involve different values of the *z*-component. As a consequence, the general unbiased moveclass must allow atom movement in all three dimensions, while ensuring that the atoms do not form multiple layers instead of the desired monolayer. But introducing constraints that limit the movement in the *z*-direction can easily lead to unphysical ‘pseudo-optimal’ atom arrangements that actually correspond to boundary minima of the global optimization problem. Frequently, such border minimum structures become unstable upon embedding in three dimensions.

Furthermore, we note that if we want to study the kinetic stability or the synthesis route of a proposed polymorph, we would strive to use only physically reasonable moves. For bulk systems or clusters, any small movements of atoms in three dimensions are feasible and encompass all allowed moves in the system. But in lower dimensions, the actual physical evolution of the system is not restricted to the one or two dimensions the system is supposed to exhibit in the end. In many cases, the actual movement of an atom from point A to point B of the low-dimensional system does not take place in the plane or on the surface of the low-dimensional system. Instead, the atom might move vertically out of the plane at A entering the gas phase or a solution adjacent to the low-dimensional system, and then move quickly parallel to the plane before inserting itself back into the plane at B. Thus, physically reasonable moves of atoms through the gas phase or via a fluid the low-dimensional system is embedded in can actually correspond to effective long jumps of atoms if projected back into the (two-dimensional) plane, i.e. if we only consider the change of the atom positions inside the low-dimensional system as constituting a ‘move’.

Conversely, if we try to model the stability of the low-dimensional structure candidates against sequences of finite atom displacements (in contrast to just verifying that the structure is a minimum of the energy function by computing the eigenvalues of the Hessian), we must distinguish between finite perturbations of the system only in the inherent (one or two) dimensions of the system, or whether we allow distortions in all three space directions after the embedding of the structure candidate in three dimensions. For more aspects of the methodology of exploring low-dimensional chemical systems, we refer to the recent literature [37].

## 3. Classes of low-dimensional systems

In general, we can divide the low-dimensional systems into several groups: pure one-dimensional and two-dimensional systems, quasi-one-dimensional and quasi-two-dimensional systems, closed surfaces and composite systems. A pure one-dimensional system consists of a finite or infinite (modelled using a finite number of atoms inside a variable periodic one-dimensional cell) chain of atoms, and analogously a pure two-dimensional system corresponds to a finite or infinite number (modelled using a variable periodic two-dimensional cell) of atoms in a plane. By contrast, quasi-one-dimensional and quasi-two-dimensional systems exhibit a finite thickness of allowed space for the atoms orthogonal to the one or two prescribed dimensions, respectively, and the quasi-one- or quasi-two-dimensional character of the system only becomes obvious in the limit of infinite chains or planes. A special but very important variation is the prescription of a general (open or closed, oriented or non-orientable—like a Möbius strip) two-dimensional surface in three dimensions, upon which the atoms are allowed to move and establish stable structures. Examples are closed surfaces, e.g. a sphere of fixed radius on which the atoms are located, or infinite cylinders, where the atoms must reside on a cylinder of fixed radius which can serve as models of carbon nanotubes; such cylinders are often also classified as quasi-one-dimensional systems.

This latter case is reminiscent but subtly different from the third class, the low-dimensional composite systems. In composite systems, we provide the system with an explicit substrate, e.g. a flat (or curved) surface of a solid material, on which the atoms or molecules or clusters are placed and are allowed to move, such that energetically optimal multi-atom/multi-molecule arrangements can be identified. A special case is the deposition of a single molecule or a cluster onto the substrate, where the structure prediction refers to the possible stable shapes of the molecule or the cluster on the surface. A major difference to the case of atoms being restricted to an abstract cylinder surface is the (spatially varying) interaction between the individual substrate atoms and the deposited atoms. This can strongly influence both the energies of the possible atom arrangements on the substrate and the growth kinetics, thus allowing the generation of metastable bulk phases on the substrate through heterogeneous nucleation and growth processes. Furthermore, we note that the atoms of the substrate can also react to the presence of the deposited atoms or molecules and thus move out of their original positions into new equilibrium positions of the substrate + deposit system—in the extreme case (if the atoms are allowed to move freely during a global optimization), these atoms will leave the substrate and become part of the layer on top of the substrate (and, conversely, atoms of the deposit will enter the substrate) [53].

## 4. Examples

In this section, we are going to present some prototypical examples—a complete review of the field would go beyond the purview of this article and we refer to the literature [37,54] and references cited therein, for further examples.

### (a) Pure one-dimensional systems

As a first example of a pure one-dimensional system, we consider finite chains of silicon and carbon atoms, Si*_n_*C*_m_* of various lengths *n* + *m* ≤ 15 [37]. As energy function, we employ DFTB, as implemented in the DemonNano algorithm [55], and the global exploration was performed using simulated annealing with periodic stochastic quenches, where jump moves were allowed, as implemented in the G42 + global landscape exploration package [56]. The structure candidates found during the global search were first optimized by gradient minimization in one dimension, followed by a small perturbation of the atom positions and gradient minimization in three dimensions.

**Figure 1.**
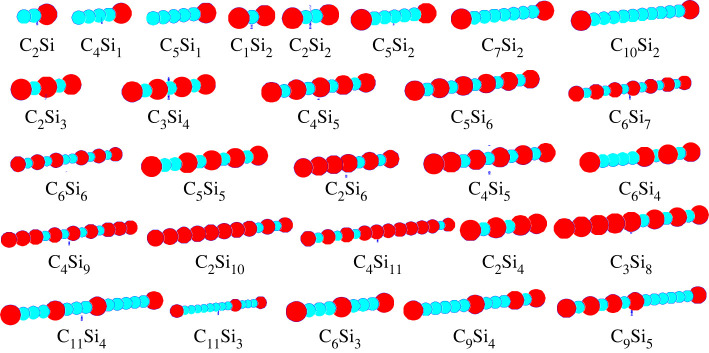
Typical examples of global or very low-energy minimum structures for a large variety of one-dimensional silicon-carbon chains (after final minimization of the DFTB energy in three dimensions) [37]. Si and C atoms are depicted as large red and small blue spheres, respectively. The first line shows chains with only one or two Si atoms; note that these are always located at the end(s) of the chain. The second line shows chains of type C_n_Si_n + 1_, where we see a perfect alternation of Si and C atoms. The next three lines show various compositions of Si and C atoms, where the chains in the fourth and fifth line possess a large excess in Si and C atoms, respectively. Throughout, we find segments with…-Si-C-Si-C-…alternations, but always with Si atoms at the ends of the chain. Note that in the interior of the chains, the minority atom species essentially always maximizes the number of nearest neighbour contacts with the majority species.

We find that the chains are stable against small perturbations after embedding in three dimensions, although we would expect the formation of Si/C rings for very long chains, where large-scale distortions of the chain in three dimensions are applied. However, we keep in mind that we are not interested in the global minimum of the system in three dimensions—this would presumably result in complex molecules or three-dimensional Si/C clusters and not in chain- or ring-like structures. Instead, here the focus is on predicting the structure under the restriction to one dimension. As ‘typical’ features of these chains, we find that they maximize the number of Si-C contacts, excess Si or C atoms form uninterrupted segments, and at the ends of the chain, we always observed Si atoms. A sample of typical global minimum or low-energy minimum chains for a number of given compositions is presented in figure 1; we note that for the longer chains, the difference in energy between structures that fulfil the aforementioned rules but differ by exchanging neighbouring atoms is very small.

A second example is the analogous structure prediction for a one-dimensional Si/C system with a variable periodic unit cell [37]. The results are similar to those for the finite chains; in particular, the global minimum for the composition Si : C = 1 : 1 was the alternating infinite …-Si-C-Si-C-…chain.

### (b) Quasi-one-dimensional systems

As an example of quasi-one-dimensional chemical systems, we mention the structure prediction for ZnO nanotubes of given diameter [57,58], and mixed Mg/Be oxide nanotubes for a variety of compositions and initial diameters [37]. Here, we focus on the mixed nanotubes in the (Mg, Be)O system of infinite length (approximated via variable simulation cells that are periodic in one dimension along the tube axis, containing 12 cations and 12 anions), where empirical damped Coulomb plus Lennard–Jones potentials were employed to approximate the energy, and simulated annealing with periodic stochastic quenches was used as global optimization procedure, implemented in G42+ [56]. Again, the structure candidates were relaxed twice, once under the constraint for the atoms to stay on the cylinder, and a second time with the atoms allowed to move away from the cylinder. For these quasi-one-dimensional periodic systems, typically some slight deformation of the cylinders occurred during the second minimization stage but all of the low-energy local minima found remained stable free-standing cylinders. In particular, even cylinders with small diameter did not collapse into a bulk-like wire structure.

**Figure 2.**
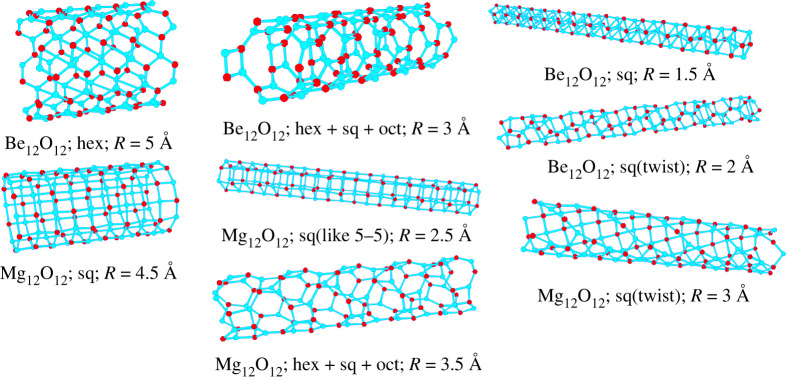
Examples of global minima or very low-energy minima of BeO and MgO on cylinders of different radii R [37]. The composition per unit cell was 12 cations (red) and 12 anions (blue), with radii ranging from 2 to 5 Å. Note that among the low-energy minima, we find straight and twisted (with respect to the cylinder axis) patterns of squares and of hexagons, and also combinations of hexagons (hex), squares (sq) and octagons (oct). As special cases, we note that for the very small radius *R* = 1.5 Å, Be_12_O_12_ looks like a cut-out of the rocksalt structure, while for *R* = 2.5 Å, Mg_12_O_12_ resembles a cut-out of the so-called 5-5 structure [59] (an ionic analogue to the hexagonal BN structure).

The global minimum structures for the BeO and MgO cylinders exhibited a threefold and fourfold coordination of the Be and Mg cations by the oxygen ions (figure 2), preferentially resulting in Be-O-Be-O-Be-O hexagons and Mg-O-Mg-O squares on the cylinder, respectively. These arrangements appeared to represent local optima of (infinite) two-dimensional structures that had been rolled up into a cylinder, analogous to the way carbon nanotubes can be visualized as rolled up graphene sheets. In most instances, these polygons are distorted after the final minimization stage.

**Figure 3.**
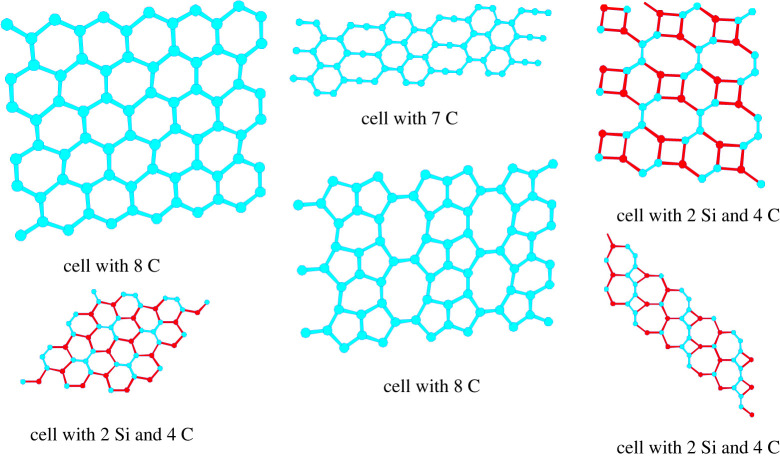
Various global and local minimum structures in the two-dimensional-planar carbon–silicon system, where we indicate the number of atoms in the variable two-dimensional periodic unit cell employed for the structure prediction [56]. For comparison, three example minima for the pure carbon system are also included. Carbon atoms are drawn in blue and silicon atoms in red. For each composition with an even number of atoms in the variable two-dimensional periodic simulation cell, the graphene analogue structure is the global minimum; two examples, one for a cell with eight C atoms and one for a cell with four C and two Si atoms, respectively, are shown on the left. Note the global minimum shown for the cell with seven C atoms. Here, the system attempts to reach the graphene pattern while obeying the constraint of only seven atoms per unit cell. We note that this structure is a minimum with rather low energy/atom and might well be realized in the experiment, e.g. by first synthesizing precursors in the shape of the double hexagon strings with one C atom attached and then reacting these to the monolayer using tools from organic chemistry; such methods for the synthesis of various covalent (organic) monolayers and polymers are an important current area of research [72–75]. The remaining three structures are local minima for cells with eight C and with two Si and four C atoms.

We note that both the squares and the hexagons can have different orientations with respect to the cylinder axis. Furthermore, combinations of different rings can be present, ranging from squares over hexagons to octagons where the latter ones only appeared in combination with other rings due to topological constraints as octagons alone cannot form a complete periodic covering of the two-dimensional plane or a cylinder surface. Many of these structures, such as the combination of 8- and 4-rings and of 8- plus 6- plus 4-rings, have also been observed in the structure prediction of periodic pure two-dimensional-planar arrangements of Si and C atoms (figure 3); similarly, some structures are reminiscent of the projection of the *β*-BeO crystal structure into two dimensions, which also reveals 4-rings and 8-rings.

**Figure 4.**
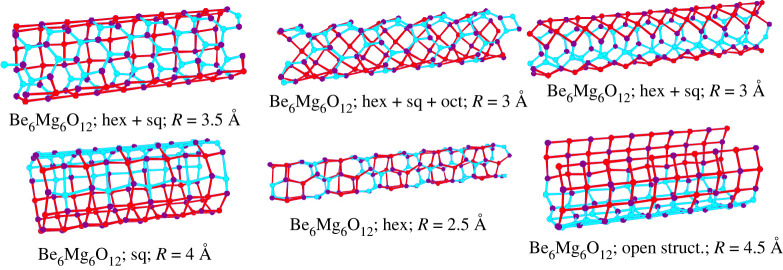
Various global and low-energy minima for Be_6_Mg_6_O_12_ on cylinders of various radii [37]. Note that square, hexagonal and mixed patterns are found. Also for some radii, we have low-lying minimum structures that leave an open patch or stripe on the cylinder surface. For small open regions, these usually close during the final local minimization stage in three dimensions, while the large ones can remain open but become very unstable already against small finite perturbations. Be, Mg and O atoms are shown as blue, red and purple spheres, respectively. In order to indicate possible distortions in the hexagons towards two highly distorted squares, all cation–anion distances up to 3 Å are drawn in the figures as red (Mg-O) and blue (Be-O) lines; for undistorted hexagons, the distance between opposite cations and anions always exceeds 3 Å.

**Figure 5.**
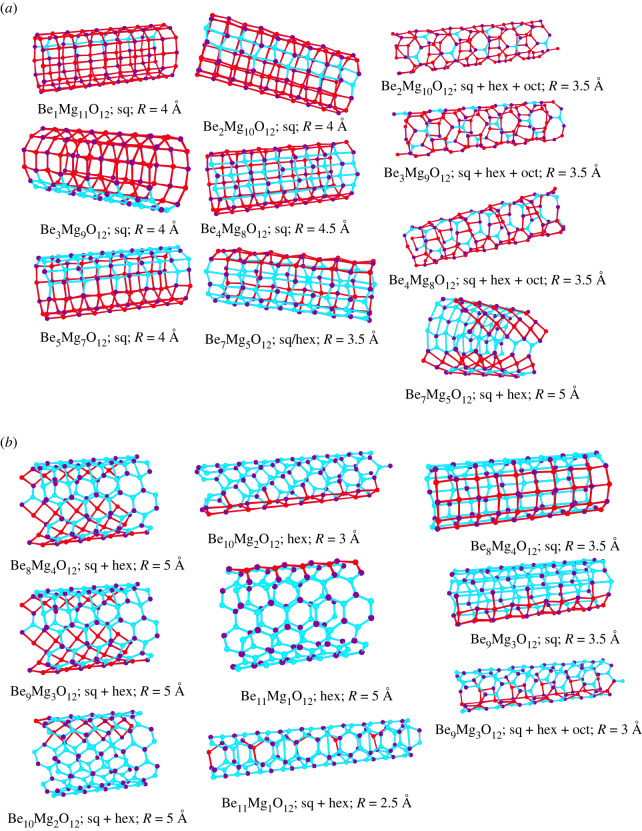
Various global and low-energy minima for Be_n_Mg_12-n_O_12_ on cylinders of various radii [37]: (*a*) *n* = 1, 2, 3, 4, 5, 7; (*b*) *n* = 8, 9, 10, 11. Note that square, hexagonal and mixed patterns (including octagons) are found. Be, Mg and O atoms are shown as blue, red and purple spheres, respectively. In order to indicate possible large distortions in the hexagons towards two highly distorted squares, all cation–anion distances up to 3 Å are drawn in the figures as red (Mg-O) and blue (Be-O) lines.

In the mixed-system Be_n_Mg_12-n_O_12_ (*n* = 1,…,11) shown in figures 4 and 5, we again observed combinations of a multitude of ring sizes. Here, we remark that, in contrast to the planar Si/C system (figure 3), we did not observe 5- or 7-rings. This is presumably due to the fact that (a) the number of atoms inside the simulation cell was not large enough, and, more likely, (b) the positively charged Be and Mg ions prefer to establish isotropic surroundings by negatively charged oxygen ions in their nearest neighbourhood. In turn, these anions try to form a dense packing on the surface of the cylinder, which does not favour odd-numbered rings. Furthermore, a cylinder surface has zero mean curvature, and thus—in contrast to a sphere (c.f. the 12 pentagons in the C_60_ molecule)—we do not need to introduce 5-rings into a perfect hexagon-network, to fulfil Euler's rule.

Another related example that falls into the purview of structure prediction is the search for the optimal distribution of (identical) charged particles that are located on the surface of a sphere, the so-called Thomson problem [60–62]. This has been studied using basin hopping as the global optimization technique. This prediction can be considered as the first step in obtaining an optimal structure of the monolayer of an ionic compound located on a sphere, where the charges employed correspond to the anions, and the cations are subsequently inserted into the holes between the anions, in analogy to the way three-dimensional crystal structures can often be described via the filling of tetrahedral or octahedral holes in a dense packing of anions or, more rarely, cations [63,64]. Since we are interested in the prediction of stable (neutral) chemical systems, we only note that the minimum energy lattice formed by the charges in the Thomson problem obeys Euler's rule with the minimal number (12) of pentagons in addition to a two-dimensional dense packing of atoms. We refer to the literature for more details [61,62], also regarding the analogous problem of optimally placing charges on more general surfaces of mean constant curvature [65].

### (c) Pure two-dimensional systems

Since it has become possible to generate free-standing monolayers of graphene, the prediction of alternative structures in monolayers of carbon and related compounds has become a topic of interest [56,66–70]. As an example, we discuss here the prediction of stable local minimum structures in two dimensions in the Si/C system using variable periodic simulation cells [56,70]. Due to the rather complex interactions present in systems containing carbon atoms that allow for many types of directional bonds in the system, empirical potentials are problematic for use in the global optimization stage, and thus, the density functional theory (implemented in Quantum Espresso [71]) was employed to compute the energies on *ab initio* level during the global optimization with the jumpmove simulated annealing module in G42 + [56]. As required, the local minima among the two-dimensional structures in the *xy*-plane were subsequently perturbed into the third dimension (*z*-direction) and re-optimized, to check whether they are stable when embedded in three dimensions (figure 3).

A multitude of two-dimensional networks consisting of Si-C rings of various sizes (3–9) were observed, which agreed with analogous ring structures in the pure C-system, many of which had also been found in a number of other exploration studies [67,68]. All the atoms in these structures had only two or three neighbour atoms, and thus, the bonds should be considered as sp- and sp^2^-hybridized bonds.

Of course, even a perfect graphene monolayer will exhibit long-wavelength oscillations in the third dimension [76,77], but we are not interested in this kind of fundamental macroscopic statistical mechanics-based instability. Instead, we focus on the stability on atom level length scales where instability would indicate that the system would greatly prefer to transform into multi-layer or bulk structures upon tiny atom displacements from the two-dimensional minimum structure. Quite generally, the instability of the embedded structures correlated with the fraction of Si contained in the compound. For the system with only silicon atoms, besides the graphene-like arrangement, only a handful of other structures were stable when atom shifts in the *z*-direction were permitted. By contrast, the presence of only a few Si atoms compared with the number of C atoms in the simulation cell usually did not lead to distortions. Only if the atom species were separated in the structure such that the carbon atoms were concentrated in one half of the cell and the silicon atoms resided in the other half, this sometimes could lead to a deformation of the Si-only region away from planarity.

Concerning the energies, we observed that the graphene analogues consisting of only hexagons of Si and C atoms were those with the lowest energy for all compositions. In order to check whether the mixed Si/C graphene-like structures were thermodynamically stable (at zero temperature), the energy of the structures was compared with the composition weighted sums of the energies of the pure C- and the pure Si-graphene modifications. The energy difference was always positive, except for the case Si : C = 1 : 1, where the Si and C atoms occupied the vertices of the graphene lattice in an alternating fashion [70]. One would therefore conclude that this specific two-dimensional SiC monolayer would be thermodynamically stable against decomposition as long as we restrict the system to two dimensions, i.e. as long as we do not allow the Si-graphene to deform into a three-dimensional bulk-like structure of lower energy. We note that attempts have been made to synthesize such a monolayer using vapour deposition methods, but they have not been successful so far (R Gutzler 2019, personal communication). An alternative approach might be the use of techniques from organic chemistry [72–75], e.g. reacting appropriate flat precursor molecules that contain alternating Si-C-Si-C-Si-C-hexagons in a fashion that is suitable for generating such a monolayer. Just recently, a successful synthesis of a SiC monolayer via heating of a silicon carbide wafer covered by a tantalum or niobium carbide layer has been reported [78].

### (d) Quasi-two-dimensional systems

Not many studies exist that deal with structure prediction in quasi-two-dimensional systems, which would correspond to either multi-(mono-)layer systems such as two/three-layer graphene systems that are of interest because of their electronic properties [79,80]. The classical quasi-two-dimensional systems would correspond to the individual layers of the so-called layered compounds such as the transition metal dichalcogenides, where the atoms inside the layers are strongly bonded via covalent bonds or ionic interactions, and the interaction between the layers is based on van der Waals interactions or the electrostatic interactions between the first non-vanishing (higher-order) electric multipoles of the layers. There exist database-based structure/property prediction studies in the literature [42], but there the computational effort is mostly restricted to performing local optimizations of given structure types on *ab initio* level and the computation of properties of interest.

The major problem with performing unbiased global landscape explorations as the tool to predict possible structures in such systems is the amount of freedom we are willing to give the atoms during the global optimization, in particular, how thick the slab is allowed to become during the exploration. Experience shows [56] that allowing atoms in a two-dimensional variable periodic unit cell to also move in the third dimension to permit the existence of structure candidates that have a finite thickness often results in a tiny two-dimensional unit cell in the *xy*-plane, of side length comparable with the diameter of an atom, and where the remaining atoms in the simulation cell stack up to form a long column in the *z*-direction! Clearly, this minimizes the total energy, because the interactions in the xy-plane are preserved due to the periodicity of the system, and the interactions in the *z*-direction are added on top.

We note that a similar problem appears when we use large (chain-like) molecules in a two-dimensional periodic variable simulation cell, which are allowed to deform into the *z*-direction. In many cases, one finds that these molecules stay with only one end-atom/group in the (*z* = 0)-*xy*-plane (fulfilling the requirement to ‘stay’ on the surface), and the rest of the molecule stretches into the *z*-direction, such that the van der Waals interactions between the molecules can be maximized. For example, instead of seeing only two neighbour atom chains along the length of the chain molecule if the molecules are forced to remain completely flat in the *xy*-plane, now each molecule can interact with three, four, or perhaps even six neighbour molecules along their full length, thus gaining a considerable amount of van der Waals interaction energy.

As a consequence, one needs to introduce (artificial) constraints—hopefully guided by the proper chemical intuition—to study and predict the structure of quasi-two-dimensional few layer systems and to identify two-dimensional patterns in the assembly of many large molecules. We note that this problem can even appear if we study the assembly of molecules on an explicit surface, where there is presumably an attractive surface–-molecule interaction, which would be lost as the molecule moves away from the surface. Nevertheless, unless the molecule–surface interaction is overwhelmingly strong, stacking of the molecules in the *z*-direction while the variable two-dimensional unit cell becomes very small can lead to a ‘thick’ quasi-two-dimensional structure with a very low energy. We note that the same problem appears for quasi-one-dimensional systems, e.g. if one allows atoms to move away from the cylinder surface, such that multi-layer tubes would be possible. Here, the energy minimization always leads us towards thick nearly-bulk-like tubes or multi-tubes, with unit cells that are periodic in the direction of the cylinder axis, where the length of the unit cell has shrunk to the diameter of essentially one or two atoms while the thickness of the atom layer deposited on the cylinder will become as large as possible for the given number of atoms in the periodic unit cell.

Therefore, the quasi-two-dimensional systems usually need to be more restricted in their configuration space and moveclass than pure two-dimensional systems, to avoid the appearance of bulk-like structures. One interesting class of quasi-two-dimensional systems derives from solids consisting of stacked slabs, such as MoS_2_ [81] or MoSe_2x_S_2(1-x)_ where the composition x can range from 0 to 1 [82]. Defining a single slab of such a solid as our quasi-two-dimensional system, we could perform a global search for the optimal arrangement of the Se and S atoms on the anion positions of a single slab of MoSe_2x_S_2(1-x)_. Since we stay with the underlying cation–anion structure–although the periodic unit cell in the *xy*-plane should be allowed to vary if necessary—an efficient moveclass for such a search would consist of atom exchanges, i.e. jump moves, followed by local minimizations. We note that the three-dimensional character of the slab provides stability when considering the embedding into three dimensions, i.e. perturbing the atoms from their positions in arbitrary directions does not really open up new options for the system; of course, a very large slab might be able to roll itself up forming a tube analogous to the carbon nanotubes [83], but for this, we need to use many more atoms in the simulation cell than we would usually employ. In this context, we remark on the difficulty of designing general robust yet fast interatomic potentials for layered structures, which are able to properly represent the energy landscape globally and not only locally, of compounds exhibiting layered structures as the global minimum. Constructing a potential that is applicable everywhere on the energy landscape for all atom arrangements of the dichalcogenide system, which also incorporates the subtle interactions that lead to formation of layers, is highly non-trivial, and many potentials that have been proposed in the past have only been suitable for very restricted regions of the landscape near the global minimum. An example for a successful design is the potential of TiSe_2_, which was employed to globally investigate the energy landscape of the three-dimensional bulk TiSe_2_ using data mining, threshold explorations and global optimization techniques [84]. We note that this problem of designing efficient, robust and globally applicable potentials is similar to the case of modelling molecules on an inorganic/metallic substrate, where completely different types of interaction potentials are needed to describe the covalent bonds inside the molecule, the metal–metal or ionic interactions among the atoms in the substrate, and the substrate–molecule interactions.

As an example system, we considered a single layer of VSe_2x_Te_2(1-x)_ for a number of compositions x [85]. Global searches for low-energy arrangements of Se and Te atoms on the anion positions of the MoS_2_-type structure proceeded via atom exchange followed by local optimizations, on the *ab initio* energy landscape level. Since the energies of the local minima identified did not vary by a large amount, it was decided to investigate the possibility of a solid solution type of behaviour, i.e. the (approximate) free energy F(x;T) = <E(x)> - TS(x) = <E(x)> - T(−xln(x) - (1 − x)ln(1 − x)) was evaluated as a function of composition x and temperature T. Here, <E(x)> was the energy per atom averaged over many different arrangements of the Se and Te atoms, and S(x) was the ideal entropy of mixing [30,86]. A more refined approximation of the free energy would include vibrational degrees of freedom, but these corrections are usually only of minor importance since the contributions of the phonons to the free energy of the pure and mixed compounds differ only slightly [86]. The result showed the presence of a miscibility gap in the quasi-two-dimensional system, with a critical temperature of approximately 500 K [85].

### (e) Composite systems

Finally, we turn to the low-dimensional composite systems. This category encompasses the (mono)layer of atoms on the surface of a bulk material, single molecules and clusters on metal or insulator surfaces, and the formation of structures in monolayers of molecules and atoms on various substrates. A further class of systems would be the formation of structures by molecules or atoms that have been intercalated inside a layered material, or inside a zeolite or metal-organic framework. The major challenge in these systems compared with the pure two-dimensional or one-dimensional systems, or the quasi-one-dimensional and quasi-two-dimensional systems, is the large number of atoms that represent the background structure (substrate, host framework, etc.), which greatly complicate the energy calculations needed for the structure predictions.

The issue here is not only the large number of atoms involved but also a particular challenge, i.e. the fact that frequently the types of interactions inside the molecule, inside the substrate and between molecule and substrate would require three different types of empirical potentials (that are often difficult to combine!) for a simplified description of the energy during the global optimization. Sometimes it is possible to replace some of the interactions by empirical potentials or even to keep the substrate atoms in fixed positions. But in general, one will have to use *ab initio* energy calculations for the whole system as much as possible already during the global exploration stage, pushing the limits even of high-performance supercomputers.

As a consequence, the global search often needs to be structured as a multi-step procedure, with different energy functions and different exploration methods employed at each stage, just to obtain some physically and chemically reasonable structure candidates. One common approach frequently employed for the structure prediction of single molecules or clusters on a substrate consists in performing many local structure optimizations (essentially a multi-quench approximation of the global optimization) starting from a set of systematically generated initial shapes and orientations of the molecules with respect to the surface. While not being as comprehensive as a global optimization based on simulated annealing, basin hopping or a genetic algorithm (for a description of these global optimization methods, see [44]), this method generates quite sensible structure candidates for the molecule on the surface with a limited amount of computational effort.

**Figure 6.**
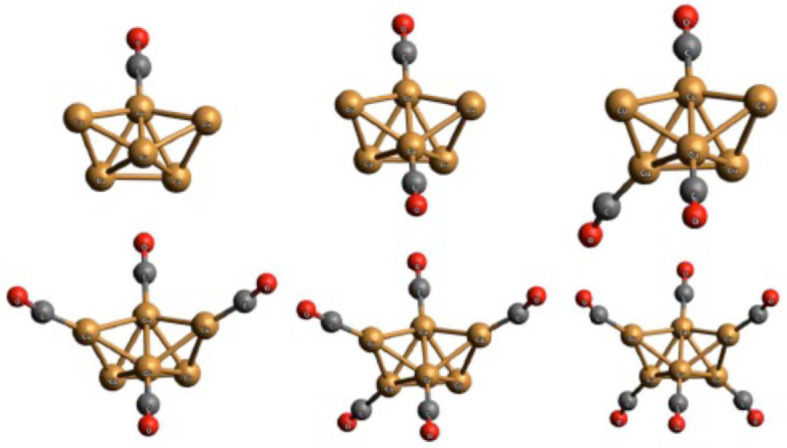
Optimal position of *n* CO molecules on a Cu_6_ cluster (*n* = 1–6); Cu, O and C are drawn as gold, grey and red spheres, respectively [89].

The study and prediction of the arrangement of small single molecules on surfaces, such as CO [87] or H_2_O [88], has been a topic of interest for many years already, and thus, we will not discuss that work in this study; a simple example is the optimal placement of CO molecules on the surface of small intermetallic clusters (c.f. figure 6) [89,90], where both the shape of the cluster and the placement of the CO molecules needed to be optimized on the *ab initio* level. In recent years, global searches for the optimal structure of small and medium-sized metal clusters placed on insulator substrates have also been performed, using genetic/evolutionary algorithms. Examples are AuCu clusters on oxides [91], PdAu and PdPt on MgO [92,93] or Cu clusters on ZnO [94].

**Figure 7.**
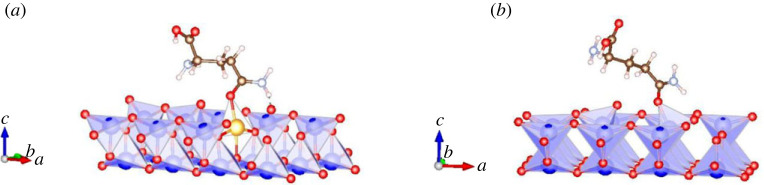
Glutamine on an (*a*) Au-doped and (*b*) undoped (001) TiO_2_ surface [95]. O, C and H atoms are shown as red, brown and white spheres, respectively, while Au and Ti atoms are gold and blue spheres, respectively. While the Au atom reacts slightly more strongly to the oxygen atom in the glutamine molecule, the doping only led to a slight distortion and re-orientation of the glutamine molecule, not to a break-up.

Another group of studies has dealt with the prediction of the shape of single mid-size molecules, in particular biomolecules on metal or insulator surfaces. However, in many of these studies, one can speak of only a limited prediction, as more or less detailed experimental data were available from the outset, or because only some sub-procedure of the global search had been performed. For a detailed discussion of the many types of methodologies employed and a review of typical examples, we refer to [54]. Here, we mention recent work on glutamine deposited on doped (with Au, Ag and Cu) and undoped TiO_2_ (anatase) surfaces, with different orientations, 001 and 101 (c.f. figure 7) [95]. Using multiple *ab initio* minimizations of pre-optimized glutamine molecules placed in various orientations near the surface showed that the doped surface resulted in many cases in the break-up of the glutamine molecule, while the deposition on the undoped surfaces never produced a break-up.

Proceeding to multi-atom/multi-molecule layers, the most straightforward one—also from the point of view of the energy functions—is the study of the rearrangement of atoms in the surface layer(s) of a bulk material [96–99]. Due to the fact that the atoms on the surface face the vacuum on one side and the bulk on the other, the optimal arrangement is no longer identical with the bulk positions, but all the surface atoms are slightly shifted. As long as this shift only occurs in the *z*-direction (the original unrelaxed surface is assumed to be the xy-plane), this effect is straightforward and often not even visible in the experimental structure determination. Of more interest are the so-called surface reconstructions, where the atoms are displaced in the *xy*-plane, leading to a different periodicity on the surface than in the bulk. The theoretical determination of the new surface unit cell can be treated as a structure prediction task, and the usual global optimization tools or machine learning can be employed [97,99]. In this situation, it is often possible to employ the same empirical potential for the surface atoms as for the bulk atoms, and thus, this global optimization can be performed on empirical potential level, with a possible refinement on *ab initio* level, if necessary. However, one needs to keep in mind that polar surfaces need a special treatment due to the appearance of (macroscopic) dipoles when the solid is approximated by a finite slab [100].

Another interesting case is the presence of only a partial monolayer of atoms of the bulk material on the surface. Now the interactions between the atoms in this incomplete surface layer, and those between the surface layer atoms and the bulk atoms compete, and various kinds of islands and unusual structures can appear. An example would be the simulation of structure formation by Zn and O atoms on a ZnO substrate, where the resulting atom arrangements were found to be in good agreement with experimental observations [100].

A more complex situation appears in the prediction of the structure of monolayers of certain types of atoms on top of a substrate consisting of different types of atoms. One example would be the structure formation of alkali halides on a LiNbO_3_ substrate (001 orientation) [101]. Here, we compared the standard square-pattern monolayer cut through the bulk rocksalt and CsCl structures commonly found in the alkali halides, with a monolayer of the so-called 5–5 structure (corresponding to an ionic analogue of the hexagonal boron nitride structure), which had been found as a low-energy local minimum during global optimizations of the NaCl system [59] and subsequently for all 20 alkali metal halides [102]. It was found that the 5–5 structure should be able to exist as a stable monolayer, and might even serve as the nucleus of a thin film of the (metastable) 5-5 modification of the alkali halide.

A similar study was performed predicting the structures of MgO single (and also double) layers on an Al_2_O_3_ sapphire substrate for a variety of densities of MgO, using empirical Coulomb + Lennard–Jones potentials [56]. The global minimum was again the square-pattern derived from the standard rocksalt bulk modification of MgO (which we have seen already as the preferred arrangement for the MgO cylinders in figure 2), both as a complete lattice and as a lattice with islands of holes. In addition, layers of BN-like hexagons were found as stable low-energy minima, and furthermore patterns, where stripes of hexagons and squares of different widths alternated; we note here that such alternations between the 5-5 structure and the rocksalt structure had also been found during early global optimizations of bulk NaCl [59]. When comparing these minimum structures with experimental studies of the deposition of MgO on Al_2_O_3_ [103], we find that some of the measurements can be interpreted as the presence of a couple of layers of the 5-5 modification of MgO. Very recently, a similar study was performed on the *ab initio* level [104], where the possible structures of NaCl films on a graphite substrate were investigated with a genetic algorithm, in combination with deposition experiments. It was found that the 5-5 structure was a low-lying energy minimum, in agreement with the results of the experiment [104].

**Figure 8.**
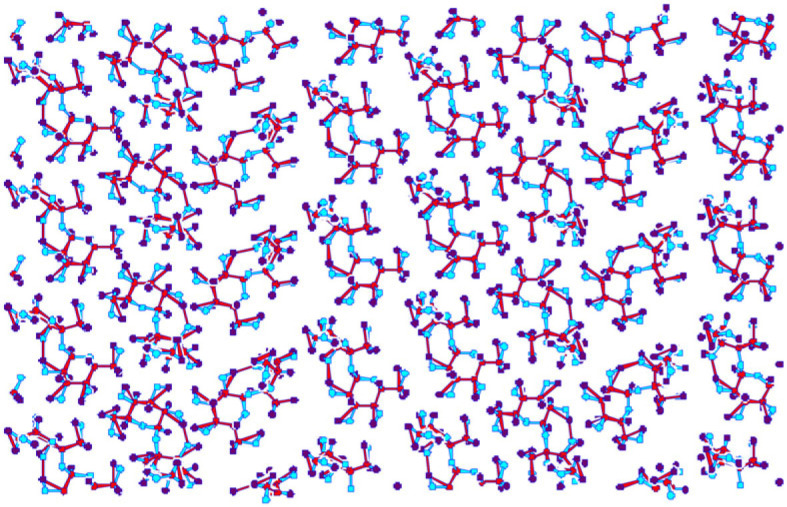
Typical low-energy minimum structure of sucrose molecules on a smooth attractive surface [54]. The shape of the individual molecule had been optimized on a Cu surface and was kept fixed during the global optimization.

As a final example, we are going to discuss the formation of multi-molecular structures on metal surfaces, for the cases of sucrose and two polymer-forming molecules (1,3,5-tris-(4-bromophenyl)-benzene and 1,3,5-tris(4-bromomphenyl)−1,3,5-triazine). For sucrose, a multi-step modelling process was employed [54,105]. In a first stage, the structure of a single sucrose molecule on a Cu surface was found using IGLOO, a global optimization algorithm. The resulting optimal structure was employed in a multi-molecule arrangement using a variable periodic unit cell and empirical molecule–molecule interactions, to find candidates for optimal patterns of groups of sucrose molecules on the surface (c.f. figure 8). The results of both the individual molecules and the periodic patterns served as starting points for fitting the scanning tunnelling microscopy (STM) data of the experiment, and good agreement was obtained [105].

**Figure 9.**
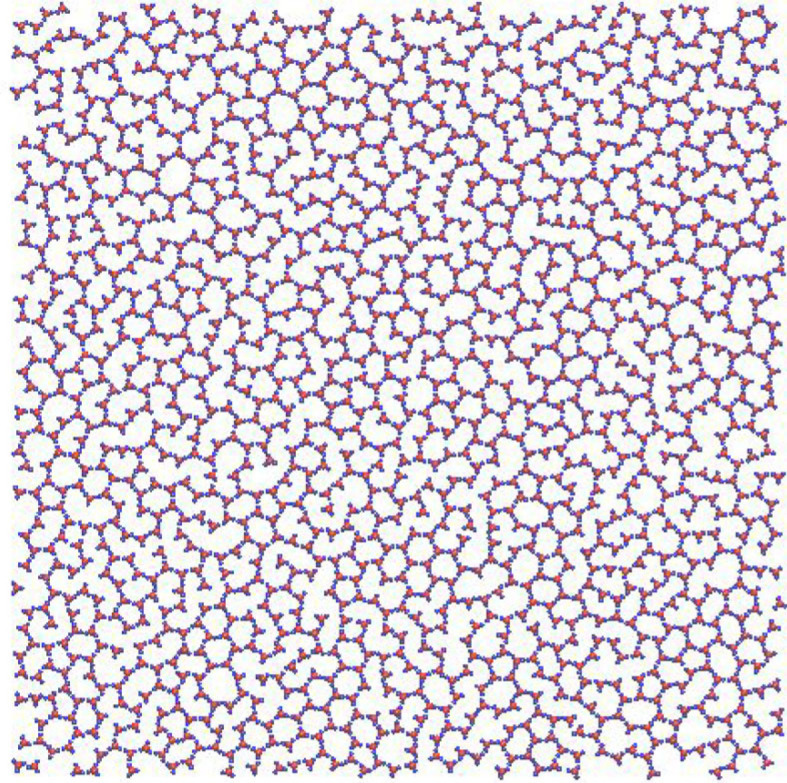
Small cut-out of the structure of a molecular dynamic simulation of approximately 5700 molecules of (1,3,5-tris-(4-bromophenyl)-benzene after approximately 240 ps [106]. The red spheres indicate the position of the central benzene ring, while the blue spheres correspond to the bromophenyl units.

**Figure 10.**
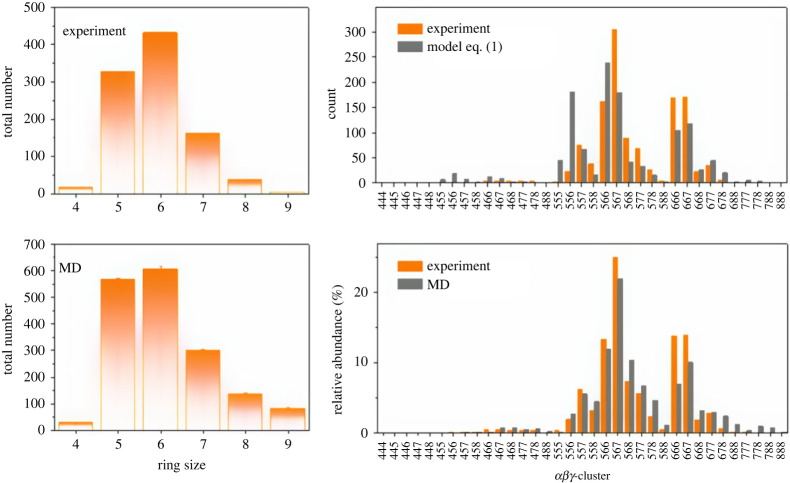
Comparison of ring-size and αβγ cluster distributions for (1,3,5-tris-(4-bromophenyl)-benzene, from experiment, molecular dynamics (MD) simulations and expected from a mean-field model for size distributions $P(\alpha ,\beta ,\gamma )=P_{\alpha }\times P_{\beta }\times P_{\gamma }\times f(\alpha ,\beta ,\gamma )$ (equation 1) [106]. An αβγ cluster corresponds to the three rings of sizes α, β and γ that intersect at a corner of the lattice, f(α,β,γ) is the multiplicity (1, for ring index ααα; 3, for ring index ααβ, with α≠β; 6, for ring index αβγ, with α,β,γ all different), and $P_{\alpha }$ is the probability of an α-gon according to the observed ring-size histogram.

In the case of the two polymer-forming molecules [106], it was observed in the experiment that the deposit did not form periodic structures but produced amorphous polymers instead. Thus, the global optimization was replaced by long molecular dynamics simulations (c.f. figure 9), which showed that both two-dimensional polymers exhibit ageing behaviour. The structures evolved on logarithmically slow time scales towards the global minimum structures of simple hexagonal patterns of the benzene and triazine molecules. Furthermore, the topological properties (ring statistics, etc.) of the experimentally observed polymer networks were in satisfactory agreement with those of the simulated ones (c.f. figure 10) [106].

## 5. Concluding remarks

The field of structure prediction of low-dimensional systems has seen much activity and great progress in the past decade. This applies both to the diversity of the systems studied and the development of search algorithms, where the similarities with and differences to the methods employed in the more established field of structure prediction of crystals, molecules and clusters are becoming clear. In particular, the embedding of the structure candidates of the ideal one-dimensional or two-dimensional system in three dimensions required for the analysis of their stability in realistic situations, as well as the presence of a background structure such as a substrate that can both stabilize and destabilize the structure found for the ideal one-dimensional/two-dimensional system, need to be taken into account when investigating the rich space of possible polymorphs of low-dimensional systems and connecting the results to real chemical systems. While trying to provide a general overview of the great diversity of low-dimensional systems for which structure prediction should be, can be, and has been performed, the focus of this work has been on the general concepts and issues involved in structure prediction of low-dimensional systems, and less on giving a complete list of all studies that have been published in this field.

Clearly, the drive towards increasingly more nano-structured materials for modern technological applications will ensure that the study of both stable and metastable low-dimensional structures in many chemical systems will continue to grow in importance. This highlights the need for the development of new techniques, or suitable modifications of old ones, for the exploration of the energy landscape of such systems and the efficient prediction of their structures. But more importantly, it points to the next stage on the road towards the *ab initio* design of such materials [107], the development of a systematic approach to model and optimize the synthesis routes employed in the actual preparation of the desired low-dimensional polymorphs that are the constituting elements of such nano-structured materials.

## Data Availability

This article has no additional data.
